# Atrial septal defect closure in children at young age is beneficial for left ventricular function

**DOI:** 10.1093/ehjimp/qyae058

**Published:** 2024-06-08

**Authors:** Pia Sjöberg, Henning Clausen, Håkan Arheden, Petru Liuba, Erik Hedström

**Affiliations:** Clinical Physiology, Department of Clinical Sciences, Lund, Lund University, Box 188, 221 00 Lund, Sweden; Department of Clinical Physiology, Skåne University Hospital, Entrégatan 7, 221 85 Lund, Sweden; Paediatric Cardiology, Children’s Heart Centre, Skåne University Hospital, Entrégatan 7, 221 85 Lund, Sweden; Paediatrics, Department of Clinical Sciences, Lund, Lund University, Box 188, 221 00 Lund, Sweden; Clinical Physiology, Department of Clinical Sciences, Lund, Lund University, Box 188, 221 00 Lund, Sweden; Department of Clinical Physiology, Skåne University Hospital, Entrégatan 7, 221 85 Lund, Sweden; Paediatric Cardiology, Children’s Heart Centre, Skåne University Hospital, Entrégatan 7, 221 85 Lund, Sweden; Paediatrics, Department of Clinical Sciences, Lund, Lund University, Box 188, 221 00 Lund, Sweden; Clinical Physiology, Department of Clinical Sciences, Lund, Lund University, Box 188, 221 00 Lund, Sweden; Department of Clinical Physiology, Skåne University Hospital, Entrégatan 7, 221 85 Lund, Sweden; Diagnostic Radiology, Department of Clinical Sciences, Lund, Lund University, Box 188, 221 00 Lund, Sweden; Department of Radiology, Skåne University Hospital, Entrégatan 7, 221 85 Lund, Sweden

**Keywords:** congenital heart defect, ventricular function, pressure–volume loops, cardiac magnetic resonance (CMR) imaging • children, paediatric

## Abstract

**Aims:**

Atrial septal defects (ASDs) lead to volume-loaded right ventricles (RVs). ASD closure does not always alleviate symptoms or improve exercise capacity, which is possibly explained by impaired left ventricular (LV) haemodynamics. This study evaluated the effect of ASD closure in children using non-invasive LV pressure–volume (PV) loops derived from cardiac magnetic resonance (CMR) imaging and brachial blood pressure, compared with controls.

**Methods and results:**

Twenty-three children with ASD underwent CMR, and 17 of them were re-examined 7 (6–9) months after ASD closure. Twelve controls were included. Haemodynamic variables were derived from PV loops by time-resolved LV volumes and brachial blood pressure. After ASD closure, LV volume increased [76 (70–86) vs. 63 (57–70) mL/m^2^, *P* = 0.0001]; however, it was still smaller than in controls [76 (70–86) vs. 82 (78–89) mL/m^2^, *P* = 0.048]. Compared with controls, children with ASD had higher contractility [2.6 (2.1–3.3) vs. 1.7 (1.5–2.2) mmHg/mL, *P* = 0.0076] and arterial elastance [2.1 (1.4–3.1) vs. 1.4 (1.2–2.0) mmHg/mL, *P* = 0.034]. After ASD closure, both contractility [2.0 (1.4–2.5) mmHg/mL, *P* = 0.0001] and arterial elastance [1.4 (1.3–2.0) mmHg/mL, *P* = 0.0002] decreased.

**Conclusion:**

Despite the left-to-right atrial shunt that leads to low LV filling and RV enlargement, the LV remains efficient and there is no evidence of impaired LV haemodynamics in children. Closure of ASD at young age while the ventricle is compliant is thus beneficial for LV function. LV volumes, however, remain small after ASD closure, which may impact long-term cardiovascular risk and exercise performance.

## Graphical Abstract

**Graphical Abstract:**
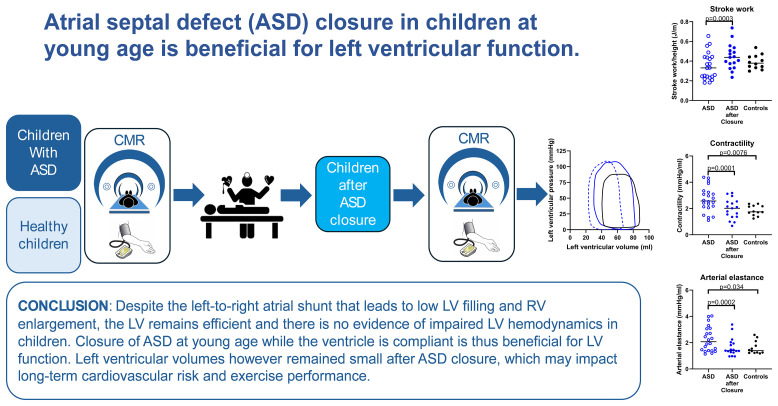

## Introduction

**Figure 1:**
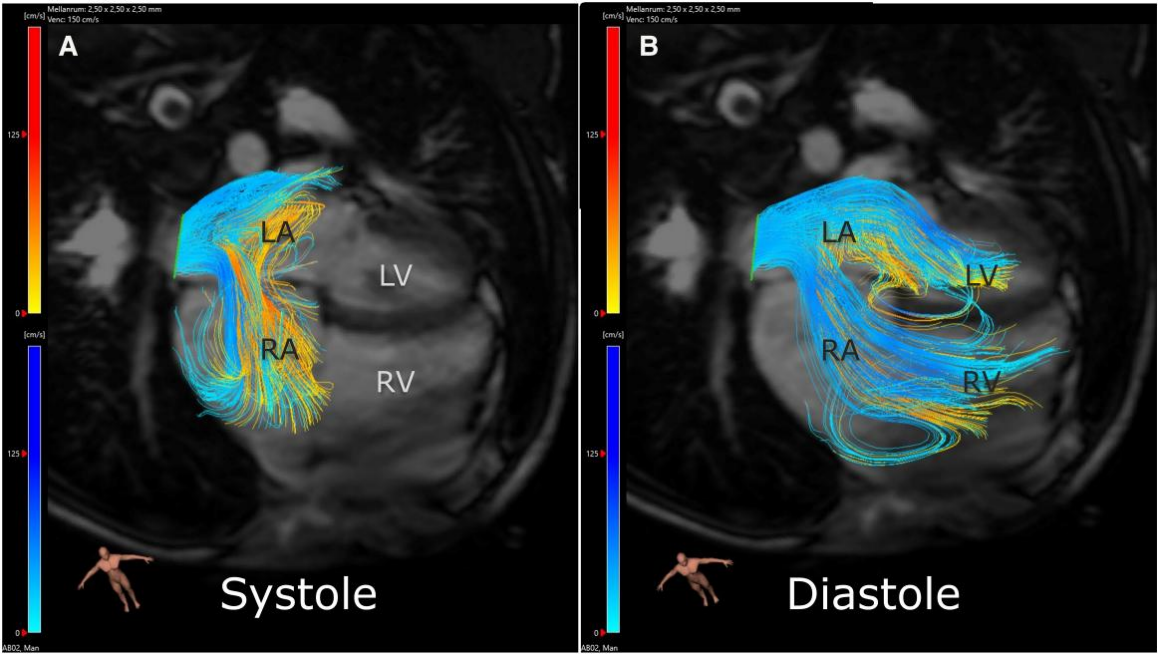
Blood flow through an ASD in a child. Streamlines from four-dimensional flow overlaid on cine images by cardiac magnetic resonance imaging. The yellow streamlines show the flow from the left pulmonary veins, and the blue streamlines show the flow from the right pulmonary veins. The left-to-right shunt is visualized in systole (*A*) and in diastole (*B*). LA, left atrium; LV, left ventricle; RA, right atrium; RV, right ventricle.

Patients with an atrial septal defect (ASD) have a dilated right ventricle (RV) due to increased volume load caused by the atrial left-to-right shunt, as visualized in *Figure 1*, but they may also have a small left ventricle (LV) due to underfilling.^1^ Closure of the defect leads to decreased RV volumes and increased LV filling.^2^ However, LV remodelling seems to be age dependent, and some patients corrected at older age show signs of diastolic LV dysfunction with associated smaller LV volumes.^3^ Little is known about long-term remodelling throughout childhood and adolescence, although survival after surgical ASD closure appears comparable to the general population.^4,5^ Adult patients with ASD may have decreased exercise capacity even late after closure, which could potentially be explained by LV dysfunction.^6–8^ The LV diastolic dysfunction^9^ with subsequent atrial dilatation may also explain the increased risk of atrial fibrillation. Atrial dilatation is however by definition of the patient’s age not longstanding in children including adolescents, why atrial fibrillation is less common.^10^

Pressure–volume (PV) loops contain information about LV function and could potentially give information about LV haemodynamics in patients with ASD. However, as PV loops commonly is an invasive procedure, clinical utility is impeded, especially in children. This has changed with the introduction of validated non-invasive PV loops assessed using cardiac magnetic resonance (CMR) imaging and brachial cuff blood pressure.^11,12^ Information on stroke work, ventricular efficiency, contractility, and arterial elastance is thus more widely available. In adults with ASD, LV stroke work is lower and LV contractility and arterial elastance higher compared with healthy volunteers.^13^ Even though ASD closure in these adults increases stroke work, neither contractility nor arterial elastance seems to normalize,^13^ and it is not known to what extent closure of ASD in paediatric patients affect short- and long-term haemodynamics.

Further, both adult and paediatric patients may have endothelial dysfunction that may improve after ASD closure, and long-standing increased sympathetic tone may play a role in vascular dysfunction.^14^ It is currently unknown to what degree RV volume loading and LV underfilling may impact reversal of endothelial dysfunction after ASD closure. Again, PV loops include information on the heart and the ventricular-arterial coupling and could thereby give insight into the mechanism of LV and vascular remodelling before and after ASD closure.^11,12,15^

The aim of this study was therefore to investigate haemodynamic variables derived from non-invasive PV loops in paediatric patients with ASD compared with controls and to assess the effect of ASD closure.

## Methods

### Study design

The study was performed in accordance with the Declaration of Helsinki (revised 2013) and was approved by the National Review Board (Nr.: 2019-05490). Written informed consent was obtained from the children’s parents, with the child’s wish to participate considered. The study is reported according to the STROBE guidelines.^16^

This prospective study was performed at a Swedish tertiary centre for congenital heart defects. The inclusion time for patients with ASD was 2 years (March 2021 to February 2023). The number of participants is based on the sample size calculation by Bellenger *et al*.,^17^ taking into account the high precision of CMR. Children with ASD under the age of 18 years, with or without partially anomalous venous drainage, referred and accepted for ASD closure were prospectively and non-randomly recruited based on the chronological order of planned intervention. Exclusion criteria were contraindication to CMR or the need for general anaesthesia to undergo the examination. All underwent CMR and simultaneous brachial cuff blood pressure measurements before intervention and 6–12 months after ASD closure. No child was examined under general anaesthesia. Sedation by dexmedetomidine at 2–3 µg/kg/dose intranasally (Dexdor©; Orion Pharma, Espoo, Finland) was used as per local clinical practice if needed. Twelve healthy paediatric volunteers, with normal electrocardiogram (ECG), no medication, cardiovascular disease, or hypertension, were also examined, all without sedation. Healthy volunteers were age-matched ± 1 year (standard deviation 1.5).

### CMR imaging

CMR imaging was performed with retrospective ECG gating in the supine position using a 1.5 T Magnetom Aera (Siemens Healthcare, Erlangen, Germany). Short-axis balanced steady-state free-precession cine images with retrospective ECG gating covering the entire heart were acquired for assessment of ventricular volumes. Two-dimensional free-breathing through-plane phase-contrast flow measurements were performed in the ascending aorta and pulmonary artery in children with ASD to assess effective stroke volume and to calculate shunt ratio (Qp/Qs) with non-invasive brachial blood pressures acquired using age-appropriate cuffs during CMR.

### Image analysis

LV endocardial borders were delineated in all time frames and analysed using the Segment software (http://segment.heiberg.se) with a validated method for PV-loop computation.^11,12^ Ventricular volumes were indexed to body surface area (BSA).

### Haemodynamic variables

Haemodynamic variables derived from the PV-loop analyses were stroke work, potential energy, ventricular efficiency, external power, contractility, arterial elastance, ventricular-arterial coupling, and energy per ejected volume. Stroke work, potential energy, and external power were indexed to height to be able to compare children of different sizes. Height was chosen in favour of BSA for this comparison, as the difference in exercise capacity found between adolescent boys and girls was reflective of difference in lean body mass,^18^ and also to avoid bias due to a large variation of body mass index.^19^

### Statistical analysis

Statistical analyses were performed in GraphPad Prism version 10.0.0 (GraphPad Software, Boston, MA, USA; www.graphpad.com). Continuous variables are presented as median and interquartile range (IQR) and categorical variables as absolute numbers or percentages. Mann–Whitney rank test assessed differences between patients with ASD and controls and Wilcoxon’s paired signed rank test assessed differences between patients before and after ASD closure. Linear regression was used to assess the impact of age at ASD closure on haemodynamic variables derived from the PV loops. *P*-values <0.05 were considered to show significant differences.

## Results

**Figure 2:**
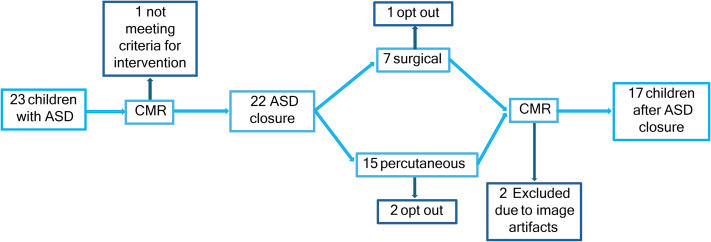
Inclusion of children with ASD.

*Figure 2* shows the inclusion chart, and *Table 1* shows participants’ characteristics. One patient was excluded from the analysis since the shunt was small and the patient did not meet clinical criteria for ASD closure. Two patients included in the study had partial anomalous pulmonary venous connection corrected at the time of surgery. Six patients received sedation by dexmedetomidine to undergo CMR.

**Table 1:** Participants’ characteristics and cardiac variables

	Children with ASD *n* = 22	Children after ASD closure *n* = 17	Controls *n* = 12
Age (years)	8 (5–13)^####^	11 (7–14)	9 (7–12)
BSA (m^2^)	1.1 (0.8–1.5)^####^	1.3 (1.0–1.6)	1.1 (0.9–1.3)
Height (cm)	137 (110–160)^####^	144 (128–165)	140 (128–163)
ASD secundum	20 (91%)	—	—
Sinus venosus defect	2 (9%)	—	—
Qp/Qs	1.8 (1.6–2.4)	—	—
Surgical closure	7 (32%)	—	—
Percutaneous closure	15 (68%)	—	—
Time from 1st CMR to ASD closure (days)	1 (1–1)	—	—
Time from ASD closure to 2nd CMR (months)	—	7 (6–9)	—
SBP (mmHg)	102 (91–116)^####^	108 (101–115)	95 (93–101)
DBP (mmHg)	67 (52–71)	59 (56–61)	60 (55–66)
Heart rate (bpm)	83 (75–94)**	77 (64–99)	75 (68–79)
Qp/Qs	1.8 (1.6–2.4)	—	—
***Left ventricle***			
EDVi (mL/m^2^)	63 (57–70)^****,###^	76 (70–86)*	82 (78–89)
ESVi (mL/m^2^)	26 (22–30)^****,##^	30 (27–33)	35 (31–38)
SVi (mL/m^2^)	32 (39–42)^***,###^	47 (40–52)	48 (45–52)
EF (%)	60 (54–64)	60 (54–65)	58 (54–62)
CI (L/min/m^2^)	3.3 (2.7–3.6)^#^	3.5 (2.9–4.2)	3.6 (3.2–4.0)
***Right ventricle***			
EDVi (mL/m^2^)	129 (111–160)^****,####^	80 (82–101)	87 (81–93)
ESVi (mL/m^2^)	55 (48–66)^****,###^	41 (34–51)	36 (35–41)
SVi (mL/m^2^)	74 (61–92)^****,####^	52 (40–56)	49 (45–54)
EF (%)	58 (54–62)^##^	53 (46–60)	57 (52–61)
CI (L/min/m^2^)	6.4 (5.4–7.5)^****,####^	3.9 (3.2–4.3)	3.7 (3.1–4.0)

Numbers are presented as median (IQR) or *n* (%). Patients with ASD before or after closure vs. controls: *P < 0.05, **P < 0.01, ***P < 0.001, ****P < 0.0001. Patients before vs. after ASD closure:

^#^*P* < 0.05, ^##^*P* < 0.01, ^###^*P* < 0.001, ^####^*P* < 0.0001.

ASD, atrial septal defect; BSA, body surface area; CI, cardiac index; CMR, cardiac magnetic resonance; DBP, diastolic blood pressure; EDVi, end-diastolic volume indexed to BSA; EF, ejection fraction; ESVi, end-systolic volume indexed to BSA; Qp, pulmonary blood flow; Qs, aortic blood flow; SBP, systolic blood pressure; SVi, stroke volume indexed to BSA.

*Table 1* shows LV and RV volumes. Compared with controls, children with ASD before ASD closure had smaller end-diastolic [63 (57–70) vs. 82 (78–89) mL/m^2^, *P* < 0.0001] and end-systolic LV volumes [26 (22–30) vs. 35 (31–38) mL/m^2^, *P* < 0.0001] and larger end-diastolic [129 (111–160) vs. 87 (81–93) mL/m^2^, *P* < 0.0001] and end-systolic RV volumes [55 (48–66) vs. 36 (35–41) mL/m^2^, *P* < 0.0001].

LV stroke volumes were lower in children with ASD before ASD closure compared with controls [32 (39–42) vs. 48 (45–52) mL/m^2^, *P* = 0.0001]. There were no differences in ejection fraction [60 (54–64) vs. 58 (54–62) mL/m^2^, *P* = 0.8] or cardiac index [3.3 (2.7–3.6) vs. 3.6 (3.2–4.0) mL/m^2^, *P* = 0.06].

After ASD closure, there was an increase in end-diastolic [76 (70–86) vs. 63 (57–70) mL/m^2^, *P* = 0.0001] and end-systolic LV volumes [30 (27–33) vs. 26 (22–30) mL/m^2^, *P* = 0.0005] and cardiac index [3.5 (2.9–4.2) vs. 3.3 (2.7–3.6) mL/m^2^, *P* = 0.040]. End-diastolic LV volumes were, however, still smaller than in controls [76 (70–86) vs. 82 (78–89) mL/m^2^, *P* = 0.048]. There was also a decrease in RV end-diastolic [80 (82–101) vs. 129 (111–160) mL/m^2^, *P* < 0.0001] and end-systolic volumes [41 (34–51) vs. 55 (48–66) mL/m^2^, *P* = 0.0003] and ejection fraction [53 (46–60) vs. 58 (54–62) mL/m^2^, *P* = 0.004].

### Haemodynamic variables

**Figure 3:**
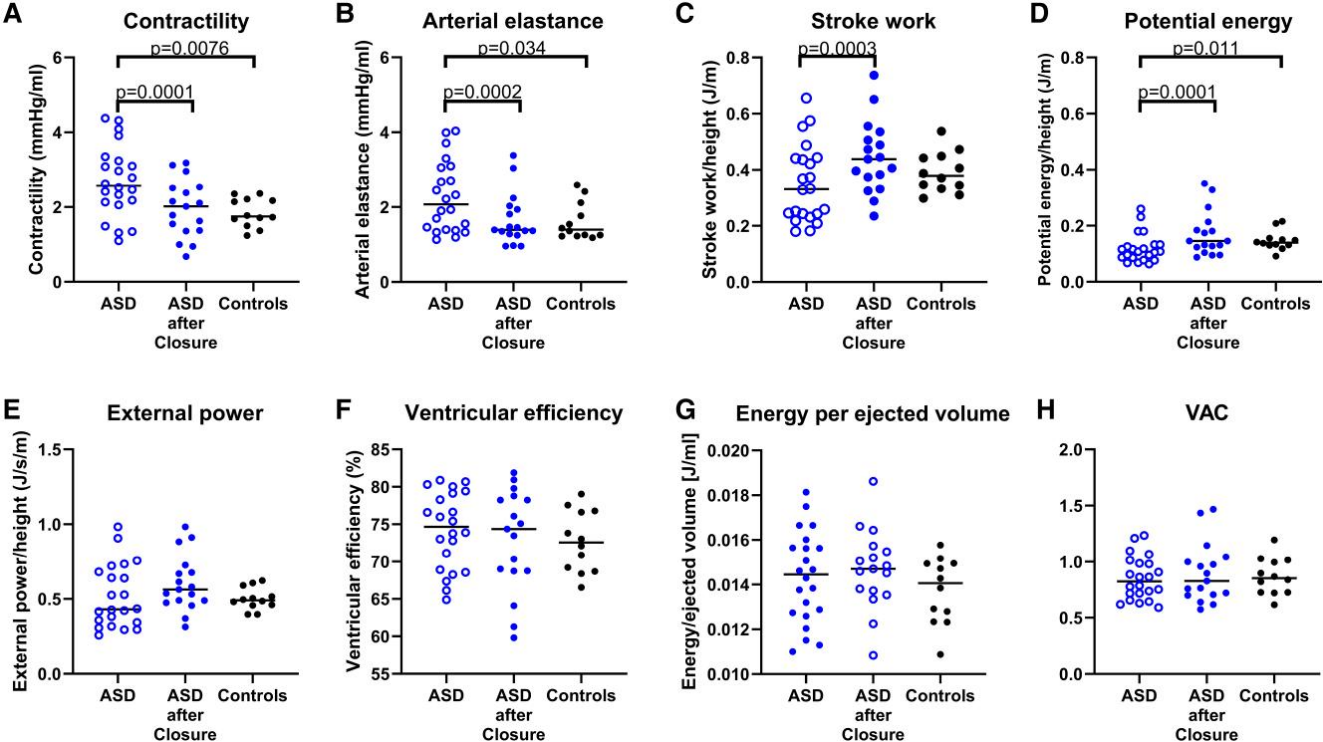
Haemodynamic variables derived from PV loops in children with ASD before and after closure and in controls *(A to H)*. Children with ASD had (*A*) higher contractility and (*B*) arterial elastance and (*D*) lower potential energy than controls. After ASD closure, there was a decrease in (*A*) contractility and (*B*) arterial elastance and an increase in (*C*) stroke work and (*D*) potential energy.

**Figure 4:**
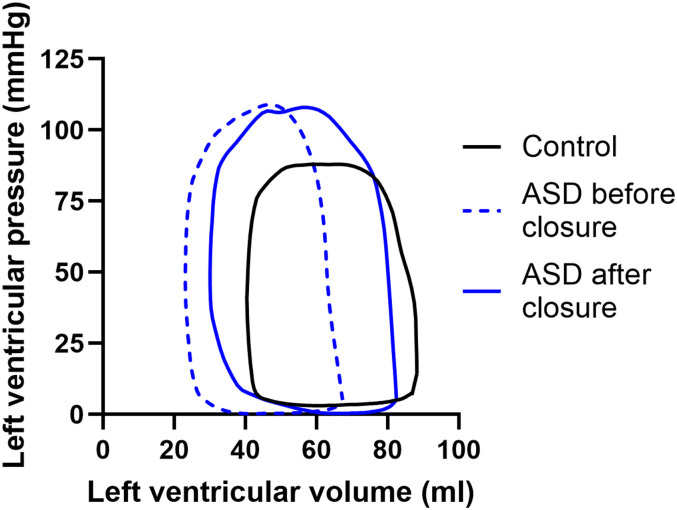
Examples of PV loops in children of the same age and height. A normal PV loop in a healthy control is shown to the right (black solid line). The PV loop in a child with an ASD is displaced to the left (dashed blue line). After ASD closure, the PV loop shifts to the right (solid blue line); however, it is still to the left of the PV loop of the healthy control.

*Table 2* and *Figure 3* show haemodynamic variables derived from PV-loop analyses. Compared with controls, children with ASD before ASD closure had lower potential energy [0.11 (0.08–0.13) vs. 0.15 (0.11–0.20) J/m, *P* = 0.011] and higher contractility [2.6 (2.1–3.3) vs. 1.7 (1.5–2.2) mmHg/mL, *P* = 0.0076] and arterial elastance [2.1 (1.4–3.1) vs. 1.4 (1.2–2.0) mmHg/mL, *P* = 0.034]. After ASD closure, there was an increase in stroke work [0.4 (0.4–0.5) vs. 0.3 (0.2–0.4) J/m, *P* = 0.0003] and potential energy [0.15 (0.11–0.20) vs. 0.11 (0.08–0.13) J/m, *P* = 0.0001] and a decrease in contractility [2.0 (1.4–2.5) vs. 2.6 (2.1–3.3) mmHg/mL, *P* = 0.0001] and arterial elastance [1.4 (1.3–2.0) vs. 2.1 (1.4–3.1) mmHg/mL, *P* = 0.0002]. *Figure 4* shows examples of PV loops in a patient before and after ASD closure and in a healthy control. There was no difference in any of the haemodynamic variables between the children who underwent open heart surgery compared with percutaneous closure before or after ASD closure (*Table 3*). The change in arterial elastance after ASD closure was affected by age at ASD closure (*r*^2^ = 0.41, *P* = 0.005).

**Table 2:** Haemodynamic variables in patients before and after ASD closure and controls

	Children with ASD *n* = 22	Children after ASD closure *n* = 17	Controls *n* = 12
Stroke work/height (J/m)	0.3 (0.2–0.4)^###^	0.4 (0.4–0.5)	0.4 (0.3–0.4)
Potential energy/height (J/m)	0.11 (0.08–0.13)^*,###^	0.15 (0.11–0.20)	0.14 (0.13–0.15)
Ventricular efficiency (%)	75 (69–79)	74 (69–79)	73 (69–77)
External power/height (J/s/m)	0.4 (0.3–0.7)	0.6 (0.5–0.7)	0.4 (0.5–0.6)
Contractility, *E*_max_ (mmHg/mL)	2.6 (2.1–3.3)^**,####^	2.0 (1.4–2.5)	1.7 (1.5–2.2)
Arterial elastance, *E*_a_ (mmHg/mL)	2.1 (1.4–3.1)^*,###^	1.4 (1.3–2.0)	1.4 (1.2–2.0)
Ventricular-arterial coupling, *E*_a_/*E*_max_	0.8 (0.7–1.0)	0.9 (0.7–1.1)	0.9 (0.7–1.0)
Energy per ejected volume (mJ/mL)	14 (13–16)	14 (14–16)	14 (12–15)

Numbers are presented as median (IQR). Patients with ASD before or after closure vs. controls: *P < 0.05, **P < 0.01. Patients before vs. after ASD closure: ^###^*P* < 0.001, ^####^*P* < 0.0001.

ASD, atrial septal defect.

**Table 3:** Haemodynamic variables in patients before ASD closure divided into those who underwent surgical closure and those who underwent percutaneous closure

	Surgical closure before ASD closure *n* = 7	Surgical closure after ASD closure *n* = 5	Percutaneous closure *n* = 15	Percutaneous closure *n* = 12
Stroke work/height (J/m)	0.3 (0.2–0.4)	0.4 (0.3–0.5)	0.4 (0.2–0.4)	0.4 (0.4–0.5)
Potential energy/height (J/m)	0.10 (0.09–0.11)	0.17 (0.13–0.27)	0.11 (0.08–0.13)	0.13 (0.10–0.20)
Ventricular efficiency (%)	74 (69–76)	69 (65–75)	77 (71–79)	77 (69–80)
External power/height (J/s/m)	0.4 (0.3–0.5)	0.6 (0.5–0.6)	0.5 (0.4–0.7)	0.5 (0.5–0.8)
Contractility, *E*_max_ (mmHg/mL)	2.8 (2.2–3.9)	1.5 (1.0–2.3)	2.5 (2.1–3.2)	2.1 (1.4–2.8)
Arterial elastance, *E*_a_ (mmHg/mL)	2.7 (1.7–3.1)	1.4 (1.1–2.1)	1.9 (1.4–3.1)	1.4 (1.3–2.0)
Ventricular-arterial coupling, *E*_a_/*E*_max_	0.9 (0.7–1.1)	1.0 (0.8–1.2)	0.8 (0.7–1.0)	0.7 (0.7–1.0)
Energy per ejected volume (mJ/mL)	16 (13–17)	15 (14–16)	14 (13–16)	15 (13–16)

Numbers are presented as median (IQR).

ASD, atrial septal defect.

## Discussion

This study shows that children with ASD have increased LV contractility and arterial elastance but otherwise seem to adjust well to the lower LV filling and increased RV filling caused by the atrial left-to-right shunt. After ASD closure, children respond well with all haemodynamic variables derived from the PV-loop returning to normal, despite smaller LV end-diastolic volumes compared with healthy controls. These smaller LV volumes may, however, have potential implications on cardiovascular risk and exercise performance as these young patients grow older.

### Cardiac volumes

Although the LV end-diastolic volume increased after paediatric ASD closure, it was still lower than in controls. In patients with RV volume load, previous focus has mainly been on right-sided heart structures, and attention has quite recently included the importance of ventricular interaction and LV function.^20,21^ Patients with ASD increase their exercise capacity on a group level after ASD closure, yet not all normalize.^22^ This may be related to altered LV function^23,24^ and has, in adults, been correlated to age at ASD closure.^6^ However, adolescents after ASD closure at young age have on a group level normal stroke volume and cardiac index at exercise, which suggests normal cardiac reserve.^25^ The clinical significance of the smallish LV volumes in some children is unknown, but one may speculate that this is part of the explanation for impaired exercise capacity in some patients after ASD closure, since a smallish heart is associated with low exercise capacity also in healthy women due to low cardiac reserve.^26^ The suggested reason for a lower exercise capacity in a person with a smaller LV is the limited ability of the heart to increase cardiac output, which is mainly due to the restricted capacity to decrease LV end-systolic volume at stress.^26^

### Energy utility

The PV loop in children with ASD before ASD closure was displaced to the left compared with controls, as related to the small LV end-diastolic and end-systolic volumes. This translates to the reduced potential energy shown in children with ASD in the current study. As blood pressure did not differ between children with ASD and controls, this also explains the lower stroke work in patients with ASD. The lack of differences both for energy needed to eject a certain volume of blood and for ventricular efficiency suggests that the LV in children with ASD adepts well despite impaired filling and the enlarged RV. LV energetics normalized after ASD closure in children in the current study, which is similar to findings in adults with ASD.^13^ Thus, the effect of ASD closure on LV energetics does not differ between children and adults, at least in the short term.

### Ventricular-arterial coupling

Another consequence of displacement of the PV loop to the left, with unchanged blood pressure, is an increase in contractility due to decreased end-systolic volume and an increase in arterial elastance due to decreased end-diastolic volume. In the current study, the high contractility and arterial elastance in children with ASD normalized after ASD closure. This differs from adults with ASD in whom contractility and arterial elastance remain high also after ASD closure.^13^ This may be explained by impaired LV compliance due to long-standing LV underfilling.^27–29^ In contrast, children seem to have a more compliant LV that adapts well to the increased volume load after ASD closure, as shown in the current study, also supported by the normalization of LV diastolic function after ASD closure in children.^30^ Increased contractility, arterial elastance, and heart rate with high sympathetic tone lead to reduced arterial distensibility and affect endothelial function.^31^ This may explain the diminished endothelial function found in adult patients with ASD and the fact that endothelial function improves after ASD closure.^14^ A high sympathetic tone may, thus, contribute to the increased risk for cardiovascular disease shown in patients with non-complex congenital heart defects.^32^ It may, therefore, be speculated that early ASD closure, and thus a shorter duration with a high sympathetic tone, might decrease the risk of cardiovascular disease.

### Clinical implications

Some patients with ASD have a restrictive LV physiology due to smaller than normal LV volumes, which could lead to lower cardiac output even after ASD closure. Attempts have been made to find a measure that can predict how the heart responds to ASD closure so as to assess the clinical risk for low cardiac output after ASD closure. In adults, LV end-diastolic volumes of <54 mL/m^2^ have been suggested to indicate this increased risk.^21^ In the current study, children with ASD did not have very small LV volumes, and none had any indication of low cardiac output. Further, low cardiac output after ASD closure is not common, and even less so in children, which makes the concept of a cut-off volume difficult to apply in practice. It also remains unclear whether this measure would be valid in children at all.

The current results suggest that even though children with ASD have a preserved LV efficiency, the smaller LV volume may lead to increased long-term risk of cardiovascular disease and lower exercise capacity due to LV functional impairment. This would support early paediatric ASD closure. Future studies should illuminate whether physical exercise programmes could increase LV volumes in patients after ASD closure or in other patients with potentially impaired LV filling, such as in tetralogy of Fallot.^26,33^

### Limitations

The control group was not matched to exact age or sex on an individual level. This is, however, unlikely to influence the results as the ranges between groups are small. The method used for non-invasive PV-loop measurement requires a non-restrictive pathway from the LV to the brachial artery. As none of the children had any aortic stenosis nor coarctation of the aorta, this should not hamper data in the current study. The volume at pressure 0 mmHg in the LV is set to be 0 mL (V0) but is likely a small positive value for a healthy LV, and method validation shows good agreement with invasive measurements.^12^ Children with ASD and low LV filling presumably have a low V0 why the approximation is deemed to be valid. The method used in this study for deriving PV loops also estimates LV end-diastolic pressure within a range of 0–15 mmHg, and this has low influence on derived variables, why this should not significantly impact the measured haemodynamic variables.^12^

## Conclusion

Despite the left-to-right atrial shunt that leads to low LV filling and RV enlargement, the LV remains efficient and there is no evidence of impaired LV haemodynamics in children. Closure of ASD at young age while the ventricle is compliant is thus beneficial for LV function. LV volumes, however, remain small after ASD closure, which may impact long-term cardiovascular risk and exercise performance.

## Data Availability

The datasets presented in this article are not readily available be due to patient data privacy concerns. Other data will be made available upon reasonable request. Requests to access the datasets should be directed to pia.sjoberg@med.lu.se.

## Lead author biography

Pia Sjöberg is a specialist in cardiothoracic surgery and clinical physiology working at Skåne University Hospital in Lund, Sweden. Her research focus is congenital heart defects, using novel MRI techniques, 4D flow and personalized computational modelling to improve the understanding of heart failure and to predict the results of interventions.
